# “For me, it is for longevity and making sure I am fit and around for my children”: exploring motivations and barriers for weight management among minoritised communities in Medway, England

**DOI:** 10.1186/s12889-024-18281-8

**Published:** 2024-03-13

**Authors:** Jennifer Teke, Obasanjo A. Bolarinwa, Lawrence A. Nnyanzi, Emma L. Giles, Louisa Ells, Scott Elliott, Sylvesters R. Okeke, Deborah O. Okeke-Obayemi

**Affiliations:** 1 Department of Nursing and Midwifery, School of Health and Life Sciences, Teesside University, Teesside, UK; 2 Department of Public Health, York St John University, London, UK; 3 Department of Demography and Population Studies, University of the Witwatersrand, Johannesburg, South Africa; 4 Obesity Institute, School of Health, Leeds Beckett University, Leeds, UK; 5 Public Health Department Medway Council, Kent, UK; 6 Centre for Social Research in Health, UNSW Sydney, Sydney, Australia; 7 ECA College of Health Science, Sydney, Australia; 8 Department of Guidance and Counselling, University of Ibadan, Ibadan, Nigeria

**Keywords:** Africa, Asia, Equity, Cost, Culture, Social support, Migration

## Abstract

**Background:**

Migration-related changes in dietary patterns and other structural and individual factors affect weight-related health practices of individuals migrating from low-and-middle-income to high-income countries. Thus, individuals of ethnically diverse backgrounds may be disproportionately affected by poorer health outcomes, including weight-related health issues. Understanding how this community could be supported to adopt weight-related healthy practices such as optimum dietary and exercise behaviour is an important issue for public health research. Against this backdrop, we explored structural and individual factors that facilitate and constrain the uptake of weight management services among members of minority ethnic communities in Medway, England.

**Methods:**

Data were collected from audio-recorded interviews with 12 adult community members from minoritised ethnic communities using a semi-structured interview guide. Participants were recruited through a purposive and convenient sampling technique. Generated data were transcribed, coded into NVivo and analysed using the reflexive thematic analytical technique.

**Results:**

Results showed that social support and health benefits of weight management were the main motivating factors for weight management among the study participants. Conversely, systemic barriers, family commitment and caring responsibilities, changes in dietary patterns post-migration and cultural norms were major factors constraining participants from adopting weight management behaviours.

**Conclusion:**

The results of this study indicate that structural and person-level factors serve as both facilitators and barriers to weight management among ethnically diverse communities in Medway, England. While our study is exploratory and opens doors for more studies among the population, we conclude that these minoritised communities could benefit from more equitable, tailored weight management programmes to support them in adopting weight-related practices.

**Supplementary Information:**

The online version contains supplementary material available at 10.1186/s12889-024-18281-8.

## Background

Individuals who migrate from low- and middle-income countries (LMICs) to high-income countries (HICs) are likely to experience a sudden or gradual change in their eating and dietary patterns [1, 2]. Malnutrition, especially at the onset of making the transition to settle in a new environment, is often inevitable for migrants [3]. Malnutrition could be overnutrition (overweight and obesity) and undernutrition (stunting, wasting and underweight) [3]. The last decade was characterised by a noticeable increase in the prevalence of obesity rates globally [4]. In the same vein, the rate of overweight reached epidemic proportions and almost outnumbered the rate of underweight worldwide [5]. Obesity is strongly linked to the incidence of type 2 diabetes, cardiovascular disease, cancers, chronic kidney disease, pregnancy complications, osteoarthritis, hypertension, stroke, and late onset of Alzheimer’s disease, amongst others [5–11].

Evidence from previous studies reveals that there is a higher risk of abdominal obesity among Bangladeshi, Pakistani, Black African and Black Caribbean migrants when compared to their white British counterparts [9, 12, 13]. Besides non-modifiable factors that can influence body weight, such as age, sex and genetic makeup, there are modifiable factors of body weight, such as diet, physical activity, and some social and environmental factors [8]. The United Kingdom is ranked third regarding the volume of sales of ultra-processed food per capita, relative to other high-and-middle-income countries, which contributes to an obesogenic society [13]. The availability of unhealthy foods has been linked to obesity more consistently than the availability of healthy foods [14].

There is consequently a need to emphasise the importance of weight management for migrants and minoritised communities in the United Kingdom because of their vulnerability, as they are disproportionately affected by weight-related chronic conditions in the United Kingdom [9, 12, 13]. Some factors motivate individuals to engage in effective weight management activities, while others act as barriers. In addition to the food environment in the United Kingdom, systemic barriers predispose migrants to unhealthy dietary patterns and activity patterns, which could contribute to the development of obesity. Though previous studies have investigated barriers and facilitators to successful weight management [15–19], it is important to stress that migrants do not constitute a homogenous group [7, 20]. Against this backdrop, the present study explored motivation and barriers against the uptake of weight management efforts and practices among minoritised communities in Medway, England. This explorative study aims to inform future studies to generate evidence to ensure population-specific and tailored interventions to help prevent and control overweight and obesity and their concomitant health problems among this population.

## Methods

### Design and paradigm

This qualitative study is theoretically anchored on the interpretivist research paradigm and reported using relevant items from the Consolidated Criteria for Reporting Qualitative Research (COREQ) checklist [21]. The study sought to explore and understand multiple perspectives from lived experience with a view to understanding how minoritised communities can be supported to engage with weight management practices and behaviour changes.

Some members of the research team, including the lead researcher, are members of the study population. Thus, this study was designed, implemented and interpreted using insider-outsider positionality. This dual positionality increases this study’s robustness and the validity of its conclusions. The insider positionality affords the research team an experiential knowledge of the study participants’ social lives and practices [22]. This knowledge, which is an asset rather than bias [23], reflexively shaped the study’s aim and questions [24] and enhanced robust conversations and culturally sensitive data collection [22].

### Participant recruitments

Participants were recruited through the Health and Wellbeing at Medway Council in Medway, Kent, England and the engagement was facilitated by the Medway Ethnic Minority Forum (MEMF) also within Kent, England. The MEMF is a charity consisting of approximately thirty registered ethnic groups which aim to promote social inclusion for minority ethnic communities in Medway by assisting them to integrate well into the host society. Two group heads at MEMF facilitated participant recruitment and advertised the study among their group members within Medway, Kent, England for volunteers to contact the research team.

Inclusion criteria for this study are those belonging to minoritised communities in Medway, that is, individuals who are not considered as belonging to the mainstream Anglo-Saxon culture or communities in Medway. For pragmatic reasons, recruitment was largely focused on individuals from African and Asian backgrounds who are living in Medway. There was no exclusion based on gender, age, class of migrant (i.e., first or second-generation migrant), utilisation of weight reduction services or any other parameter. Our overall aim was to explore the experiences of individuals belonging to the minoritised communities in Medway in relation to weight management motivators and barriers. While recruiting, we also did not intend to compare experiences across the spectrum of individuals based on socio-demographic characteristics such as a ge, gender and/or class of migrant.

Ethical clearance (Study No 117/18) for this study was granted by the Research Governance and Ethics Committee of Teesside University School of Health and Life Sciences. Participants provided informed consent before participating in the interviews. This consent also entails that participants understand their participation is entirely voluntary. This aspect of consent was stressed because of the nature of recruitment, which involves using a Charitable organisation that participants associate with. Also, consent involved participants understanding they could withdraw at any stage and this withdrawal would have no consequence on their relationship with MEMF and that the study results would be published in de-identified form in academic journals.

### Data collection method

Using a participant-oriented approach, requiring participants to share their own experiences using their own words [25, 26], data was generated using semi-structured interviews [27] to explore motivators and barriers to weight management among twelve individuals, six males and six females. Notably, experience regarding weight management in this study did not only entail having engaged in weight reduction but also perceptions around weight management [27, 28]. During the interviews, participants were asked some unidentifiable socio-demographic questions. Before delving into the interview questions focusing on the research aim, participants were asked some ice-breaking questions regarding their overall migration experiences, especially what they liked in their host countries in comparison with their home countries. The essence of these ice-breaking questions was two-fold. The first was to create a relaxing environment as participants described their positive migration experiences. These positive experiences reduced tensions and anxieties for both the interviewer and the participants. The second purpose for the ice-breaking questions was for participants to start reflecting on their experiences as minoritised groups in their host society. This set the tone for introducing the focus of the study.

Participants were asked questions regarding their nutritional and exercise practices as it relates to maintaining healthy body weight. They were also asked questions about factors that shape their nutritional and exercise practices. The interviewer used prompts and probes to guide the conversations. It is important to stress that prompts and probes were used to further understand participants’ perspectives and not as detective techniques to ascertain the veracity of their claims. Drawing from the theoretical underpinnings of this study, a semi-structured interview technique was employed to afford participants the latitude to discuss experiences they consider meaningful to the study but were not included in the interview guide (See supporting information).

Participants were also at liberty to fix the time and location for the interview based on their availability and convenience; most interviews were done either at home, workplace or at the faith temple. The interview sessions lasted between 25 and 30 min, and the number of participant interviews ended when data saturation was reached [21]. To ensure confidentiality, no identifying information was collected from participants, and a unique identification code was assigned (as shown in Table 1 and illustrative quotes).

**Table 1:** Participants’ Identification Code

Participants				
Generation	First	Second	First	Second
**Background**	Black African	Black African	Asian	Asian
**Code**	FBA	SBA	FA	SA

*NB The participants were assigned numbers sequentially from 01 to 12. Hence, at the end of a quotation, ‘SBA 12’ denotes second-generation Black African and participant number 12*.

### Data analysis

The interview sessions were audio-recorded and transcribed verbatim for the purpose of analysis. Analysis was informed by the reflexive thematic analysis (RTA) framework [22, 23]. The choice of this analytical technique was informed by its flexibility to allow a researcher’s subjectivity in producing knowledge [23]. This technique has also been used in studies involving minoritised communities of diverse ethnicities [13] to explore similarities and differences [29, 30] in the perspectives and experiences of participants based on different ethnic backgrounds [22]. The analytical technique involved a largely data-driven and bottom-up approach to reflexively generate themes from the data transcripts [22]. The analytical process began with familiarisation with the dataset from the data collection stage. The analysis was conducted by JT, who also interviewed the participants, with inputs from other members of the research team who are senior researchers with high-level expertise in qualitative research. The interview sessions were transcribed verbatim and read multiple times for familiarisation and immersion [30]. Having gained a better understanding and underlying meaning conveyed in the data beyond semantic representation [24], the transcripts were classified into codes using NVivo. The coding strategy involved inductive and deductive approaches, grounding the codes in both generated data and relevant literature [31]. Themes were generated from these codes through searching, categorising and refining [23]. Participants with similar responses relating to any reflected themes were excluded from the study’s results to avoid over-saturation [21].

## Results

### Sociodemographic characteristics of participants

Twelve individuals, six males and six females, participated in the interviews. As shown in Table 2 below, there were six participants from Asian backgrounds and six from Black African backgrounds. Ten of the participants were first-generation migrants, while two of the participants were second-generation migrants. Of the two second-generation migrants, one was of Asian background, and the other was of Black African background. The age range of the participants was 26–65 + years. All 12 participants expressed personal weight-related concerns.

**Table 2:** Sociodemographic characteristics of participants

Sociodemographic Variable	Number of participants
Age	
26–35	2
36–45	5
56–65	1
> 65	4
***Gender***	
Male	6
Female	6
***Ethnicity***	
Black or Black British-African	6
Asian or Asian British-Indian	6
***Occupation***	
Intermediate Occupation	5
Managerial and Professional Occupation	2
Unpaid Voluntary work	1
Retired	3
Home Carer	1
***Weight Status*** (Concerned about excess weight)	12

### Identified themes for motivation and barriers against weight management

#### Motivations for uptake of weight management programmes

Analysis of the experiences and perspectives shared by participants yielded two themes on motivations for weight management uptake: social support and health benefits of weight management.

### Social Support

Experiences and perspectives shared by participants indicate that social support from peers and family members motivated participants to access or adopt weight management services and/or behaviour changes. Specifically, some participants stressed the importance of gaining social support from peers who share the commonality of attending weight management services together. Participants noted that sharing this journey with peers who also wish to manage their weight was a major motivation:

“*Sometimes with this weight management, exercise or anything related, if you are doing it alone, it really doesn’t work. At least if there are two or three of you from the community, you tend to encourage each other*” *(FA01- Female).*

However, based on participants’ perspectives, gaining such social support and encouragement from a group would only work if members of the group relate together and share a similar target – weight management:

*“…is it going to be with a group of people I can relate with because having something individual would not help me now. It will be probably something that I know we have got a target, and it is not just me in this situation, I think that would help me” (SBA10 - Female).*

Furthermore, participants’ experiences indicate that aside from peers, family concern and support are important motivations for weight management. For instance, a second-generation Black African migrant tied her motivation for weight management uptake to motivation drawn from her children’s concern about her weight:

*“…my children keep telling me, ‘mummy, you have got so many flaps’, and it will be very encouraging for them to see that the flaps are gone, and mummy looks healthy and can wear things that are nice and not flabby” (SBA10 - Female).*

### Health benefits of weight management

Perspectives shared by participants indicate that they have adequate knowledge of the health risks of being overweight. For instance, some participants demonstrated good knowledge of the health consequences of being overweight. Perspectives and experiences shared by participants also demonstrate that they understand and appreciate the place of weight management in preventing these health problems:

People have problems with heart attack, diabetes and everything, and it will be better if they can help us.

“…*weight management means at least you should be eating the normal diet, do exercises and at least maintain your weight because there are so many health risk factors that are associated to weight gain like diabetes” (FBA01- Female)*.

Consequently, participants reported uptake of weight management to prevent these health problems and ensure a longer life span:

*For me on this path of weight management, losing weight is for my own future, the longevity of my own life and making sure that I am fit and am around for years to come for my children. When I think about it, it encourages me even more, to go ahead, to go for this (weight management)*” *(SBA10 - Female).*

Interestingly, the experiences shared by the participants indicate their intentionality and purposefulness in weight management uptake to avoid the negative consequences of excessive weight while also enjoying the health benefits of a good weight for their height:

“*I noticed I was getting a bit tired, so I said I had to do something about the tiredness; I used to play football, so I know I can be extremely fit, and when I put on weight, I knew this is not the right way, so that’s when I started to think hard about it (weight management)’*”*(FBA11 - Male).*

*“For me, it is also the health, not being able to walk far, not being able to do certain things, and I think personally, the contributory factor is my weight. I want to be healthy; I want to be able to walk up the staircase without feeling breathless… (SBA10 - Female).*

### Barriers

Participants expressed different views on the perceived challenges to access and uptake of weight management programmes. Four themes were identified from the perspectives and experiences shared by participants:

- Systemic barriers.
- Family commitment.
- Migration-related change in dietary pattern.
- Cultural/religious norms around weight gain and loss.

### Systemic barriers

Experiences shared by participants under the systemic barrier theme include language, food labelling, neighbourhood safety and economic challenges. For instance, some participants identified language as a major barrier to the uptake of weight management services. When such services or the health benefit of weight management uptake is conveyed in English while neglecting the dominant community languages of minoritised groups, uptake of such services and engagement with the message will be affected. This is particularly the case for first-generation participants. In describing this experience, a first-generation migrant noted:

“… *language, you know, if you want people to do something, they first have to understand what you are asking them to do, otherwise, they don’t know. Like here, you put leaflets to them in English, they don’t read, and they look and say “it’s of no use”… but if you send in Gujarati, then people read. What profit for them is health, yeah, and they are interested to read; otherwise they say oh! Put it in the bin, then it’s gone, no use. If it is in Gujarati, they read, they think about it and they ask someone to take them to the service*” *(FA05 - Female).*

Similarly, the sole use of English in weight management support services like the gymnasium could also discourage members of minoritised communities from engaging in such services. Even when they do, the language barrier could also affect the extent to which they could benefit from the services, as succinctly captured by one of the participants:

*“In the gym, the language of instruction is English. For me, it’s not a problem but for many people from the Asian background in this community, it will be difficult to follow the exercise instruction in English” (FA09 - Male).*

Another system-level barrier the participants shared was the lack of food content labelling for minoritised groups’ traditional food and delicacies. This lack of labelling limits the extent to which community members can make healthful food choices. The impact of poor nutrition labelling affected most participants, regardless of their ethnic backgrounds or generational differences. A participant of African descent clearly shared this experience in one of the interview sessions:

“*Those of us of African background, the food we eat, what we buy from the super market and stuff, they are labelled with how many calories, how many sugars they got [but for] African food, [they] are not labelled. So, what I will love is maybe if… you might not labelled* [sic] *them but at least have that information to say this kind of food is having this [a]mount of calories” (FBA04 - Male).*

Further, some participants cited the high cost of gym registration as a barrier to their intention and willingness to uptake weight management support. Accordingly, some participants stressed that gym memberships are unaffordable, and membership contracts are not flexible enough to allow for rolling contracts. Sharing this experience, one of the participants noted:

*“… all the gyms around was only on a one-year contract, there was no other way. If you are supposed to join, you have to join on a one-year contract. Now at least there are some that you can join on a monthly or whenever you can and are more flexible. Something like a rolling contract. There are not so many if any 24-hour gyms around. This might benefit me. I don’t think there’s any in Medway.” (FBA01 - Female).*

Even though some participants admitted that they do have a gym membership and could afford it, they acknowledge that lack of money could be a barrier for other individuals:

*“I pay for the gym because I can afford, many other people they are overweight and cannot afford gym membership. May be free gym membership for people who are overweight”. (FBA11- Male)*

Although participants can still engage in personal exercises without paying gym subscriptions, safety and security concerns pose additional challenges. As a result, some participants identified safety concerns in their neighbourhood as a barrier to weight management. Perspectives and experiences shared by some of the study participants indicated that work and other daily activities could impact this further, as safety was a concern when exercising early in the morning or late at night. In sharing this experience, a female-identifying participant noted:

“*…If you want to go out running, sometimes you have to think about the crime around this area. You cannot just go out running after 10pm or you cannot just go out too early because it’s a bit too scary. There’s also that restriction as to when you can safely go out” (FBA01 - Female).*

### Family commitment

Some participants discussed their commitment to their family as a barrier to weight management. Some barriers were experienced around childcare, which was common among female-identifying participants:

*“…As a single parent, I cannot really go and do exercises. It means I have to have somebody at home who can stay with the baby before I go out…Childcare is a challenge because I have got no options. If there is nobody at home to be with the child, it means one is grounded” (FBA01 - Female).*

The experiences of partnered parents also is similar as some participants who are not single mothers also shared how caring responsibilities potentially affect their engagement with weight management programmes:

*“…I have got two young children, so the sessions that I would need to attend, if I wanted to attend would need to be maybe on the weekends, maybe in the evenings when my husband is around to look after the kids. So, for me those kinds of things will definitely make a difference*” *(SA12 - Female).*

“*The main challenge honestly for me is time… time. If the programmes are run in the evenings, I am fine but during the day I am quite busy especially with the kids, family, work and other things generally” (FBA03 - Female).*

Perspectives shared by some participants showed that aside from child-caring responsibilities, commitment to family members back in home countries also contributed to weight management barriers. Experiences revealed this was two-fold: (1) in terms of the impact this had on available time and (2) the impact on the affordability of healthy food and exercise, like using the gym, when saving up to financially support family back home. This experience is captured by one of the participants:

*“Our lifestyle is ever on the go because we have caring responsibilities for our families back in Africa. In fact, we have come here to work. What time do we have to cook vegetables…and chicken curries when you’ve got people back home waiting for school fees? Are you gonna go to the supermarkets and start choosing low fat yoghurt which is twenty pounds more…low fat things are very expensive. So, if you go to the shops are you gonna be looking for that? When your family back home is waiting for money? Talking about going to the gym?…what time do I have to go to the gym whereby instead of spending three hours in the gym, I could have been earning thirty pounds somewhere (laughs)” (FBA01- Female*).

### Migration-related change in dietary patterns

The results of this study indicate that migration-related shifts in dietary patterns also affect weight management. Specifically, some participants narrated experiences of the availability and affordability of certain energy-dense foods in the United Kingdom. These readily available and affordable foods resulted in the potential for overconsumption. In narrating this experience, one of the participants of Black African descent stated:

*“Where we are coming from, certain food is not always available. Most of us here are not from wealthy backgrounds. We come from struggling backgrounds where eating things like cheese and chocolate was a luxury. When we come here (UK) and see these things readily available and affordable, we binge on it, and to get out of that habit is not easy” (FBA01 - Female).*

Another change in dietary patterns reported to affect weight management is meal time and type of food eaten. Some participants mentioned how eating at odd times due to work pressure and night shifts affected their eating patterns and the potential impact this new eating habit has on their weight management:

“*… it is difficult to maintain due to our lifestyle, eating late at night, sometimes the nature of shift work and eating at inappropriate times; I used to eat late. I have cut down on carbohydrates and am trying to be more healthy, be more conscious of the food I consume, but this is a challenge” (FBA03 - Female).*

### Cultural and religious norms regarding weight gain and loss

Experiences and perspectives shared by participants also suggest that cultural and religious norms about weight gain and loss contribute to barriers to successful weight management. Some participants mentioned that home background and sociocultural relationship with food may impact weight management. For instance, food is a form of hospitality and social bonding. Therefore, food refusal is frowned upon and considered an unkind gesture in some cultures, which could result in overconsumption.

*“…food occupies a prime place and considered as a form of hospitality, therefore refusal to eat what is on offer or offered in these gatherings is seen as a sign of ‘rudeness’ and not adhering to the cultural norms” (FBA01 - Female).*

Another sociocultural narrative that resonated among some participants is the perception that being overweight is a culturally acceptable norm. For most interviewees of African descent, being overweight is culturally perceived as a sign of good health and wealth. Potentially, this perception acts as a barrier to weight management, especially for African women who are often under pressure to have a certain body figure to gain societal appeal and acceptance. One participant stressed this perception:

*“…It’s the culture; a fat man is a sign of wealth and good health in Africa. That’s why no African man of the older generation will encourage their woman to lose weight…no. Tell me, how many African men will marry a very slim woman? Or encourage their women to lose weight? All the men are interested in the nice big ‘bum’. This makes African women put on weight because that is what the men like. For me, each time I have to go home, I have to start taking ‘ensure’ (nutritive milk drink) and also giving my children ensure and other fatty stuff so that we will look big and healthy. Slimming down is often associated with suffering and ill-health” (FBA03 - Female)*.

Another participant mentioned HIV and AIDS as health issues associated with weight loss. According to the participant:

*“if I lose weight now, all of my friends and family will be asking what is going wrong? And you know back home we used to diagnose AIDS by just looking at the way a person suddenly loses weight (she laughs)…” (FBA01 - Female)*.

In the same vein, some participants indicated that religious belief could also be a barrier to weight management. In some religions, it is inappropriate for a male health professional to examine or work with a female patient for weight management. In describing this perspective, one participant noted:

*“…another cultural thing is that women don’t particularly… especially within my community, they don’t want men to be giving them this kind of advice. You know even for weighing them or stuff like that it would have to be women. So that would be definitely another factor. Not realising that there is that segregation or cultural barrier between men and women. So that’s the other thing” (SA12 - Female).*

## Discussion

The results of this study reveal the importance of group support for a person who is overweight. Such support could be from family members, community members, peers, and mates in weight management programmes. Migrants are more likely to settle in neighbourhoods which are characterised by varied multicultural environments [19].

Our study’s results showed that most participants were keen on enjoying the benefits of weight management programmes, and they derived their motivation also from considering the potential benefits of participating in weight loss programmes. This result supports the conclusion of Wirth et al. [32], who noted that people who are overweight can be strongly motivated by the health (for better health outcomes) and social benefits (to look good) of losing excess weight. The perceived benefits of participating in a weight management programme (i.e., a person’s beliefs about whether the recommended lifestyle practices will lessen the risk of the impact of excessive weight gained) through dieting and physical activity can improve the likelihood that people would get involved in such programmes [24]. Thus, the decisions of migrants to lose excess weight could be based on their perception of the benefits and the effectiveness of the weight management programme to help them achieve their need to lose weight [17].

The study’s results suggest that a major systemic barrier faced by migrants as they seek to attain effective weight management may have to do with language and cultural barriers imposed by the host society systems. Ojo et al. [13] reported that in England, there is a low level of acceptance and a high level of underutilisation of the United Kingdom government’s healthy eating dietary resources partly due to both language and cultural barriers. There is no gainsaying the fact that effective communication is pivotal in health promotion. Poor communication with health workers and the entire health system is a common experience of migrants, especially those who are not native English speakers [27, 28]. Thus, part of the barriers minoritised communities may face in adopting weight management practices could be language and cultural barriers, as the result of our study suggests.

Furthermore, our results indicate how vulnerabilities related to survival and settling impact weight management practices among participants. In support of previous studies among migrants in high-income countries [9, 13, 32], we found that some of our participants reported how struggles with busy lifestyles and work schedules impact their nutritional practices. Busy work schedules impact food preparation, thereby making some of these migrants settle for foods that are readily available or easier to prepare in terms of preparation time, even if they are not considered healthy [13].

In addition, in line with previous studies [9, 32], we also found that some of our participants may consider the time to engage in weight management practices a waste as they could be earning. Therefore, there is the vulnerability of committing more time to work instead of going to the gymnasium or engaging in physical activities. This is partly to enable these migrants to make more money to meet the financial commitments they have for their families in their home countries. Our results also showed how financial commitment to families back home impacts the choices that some of our participants make in selecting food choices. In line with the conservative norms of communality that are prevalent in African and Asian countries [22], some of our participants reported settling for cheaper and less healthy food products to enable them to save and send money back home to assist their families. This result has implications for tailored health education to encourage members of these communities to assist their communities back home, but also to find a balance as their health and well-being are important for them to continuously support their families in their home countries.

The study’s findings revealed that past food insecurity is why parents permit their children to overindulge in unhealthy food consumption in their host country. This is in tandem with the report of previous studies by Anderson et al. [33], Alsubhi et al. [5], and Amoah et al. [29]. In some instances, there could be overindulgence in unhealthy foods, particularly in the early months or years of arrival in a high-income country like the United Kingdom, because of the excitement of now living in a new society with an abundance of food items that are not commonly eaten in home countries [31]. The high availability of processed food, fast food, and canned food, which are often unhealthy due to their caloric content [5], may become a major contributor to weight-related issues for these migrants. Consequently, it is important to ensure that members of these communities are sensitised, especially upon arrival, to the need to ensure a healthy diet and other activities for weight management, considering that migrants are disproportionately impacted by chronic health conditions in the United Kingdom that are diet-related [5–11].

Moreover, our study’s results also show how cultural norms influence dietary patterns and weight management efforts. Food’s social and cultural meaning to migrants could hinder effective weight management. The traditional meaning of food, which many migrant groups acknowledge, transcends the mere supply of energy as these migrants attach both social and cultural meanings to food, which is viewed as a part of their family heritage and a source of memories from their country of origin [13]. This is often why an individual who aims at attaining effective weight management may be frowned at for rejecting such traditional foods with high calories [34].

Our study’s results also indicate there is a cultural perception, especially among migrants from Africa, of people who are overweight as people in good health and wealth. This may bring about a cultural clash being experienced by migrants of African descent in realising that in England, it is common for women to be cautious of their weight, unlike what obtains in their home countries, where there is the societal appreciation of women who are thick and curvy, thereby making it absurd for women to aim at reducing excess weight [31]. As a result of this cultural perception of weight gain, migrants may not prioritise weight management as their cultural norms may not encourage weight loss [9].

Also, there is the cultural perception of overweight, especially among women, indicating high social class and evidence of fertility [7]. Thus, since large body sizes are, collectively, a cultural symbol of wealth, beauty and fertility [5, 7], migrants from traditional cultures where this norm is common may indulge in consuming food products that may be obesogenic as well as limit physical activities to preserve or achieve weight gain.

Finally, the results also indicate religious norms and beliefs may also impact weight management practices among some participants. In support of previous studies [5], some religious beliefs may influence the adoption of lower levels of physical activity and dietary patterns, which may be unhealthy. Some of these beliefs may involve rules for men and women as regards participation in physical activities. Against this backdrop, making physical activities, including gym attendance, more equitable and culturally sensitive in Medway could involve taking cognisance of these cultural beliefs to encourage members of minoritised communities who hold such beliefs to engage in weight management practices.

### Strengths and limitations of the study

To the best of our knowledge, this study is the first to explore facilitators and barriers against weight management among ethnically diverse communities in Medway, England. This study, therefore, provides an important insight for further studies and evidence to design equitable and culturally sensitive weight management service provision and support for minoritised communities in Medway. This study was designed, implemented and interpreted using insider-outsider positionality as the research team comprises insiders and outsiders vis-à-vis the population of interest. This dual positionality increases the study’s robustness and the validity of its conclusions. Therefore, this study provides high-quality empirical insight that could guide future studies and interventions on how this priority population for weight-related health problems [5–11] can be supported to protect and promote their health.

These strengths notwithstanding, the results of this study need to be understood with some caveats. First, the study’s results may not be generalised, considering it was a qualitative study and involved a small sample, though qualitative studies do not aim at generalisability. Second, the strategy for recruitment, though pragmatic, may have opened up recruitment likelihood to particular members within the overall study population while shutting out others. In the same vein, since data collection was in English, it is also likely that participants who are not comfortable or confident speaking English may have refrained from participating due to the language barrier. Therefore, this study is only a gateway and not conclusive in relation to understanding myriad factors impacting minoritised communities’ engagement in weight management practices.

### Implications and recommendations for future research

The results of this study have implications for public health research, policy and practice. In relation to research, future studies need to build on the results of this study. This way, we can better understand how tailored interventions can be designed and evaluated to support this population group to adopt weight-related healthy practices, avoid weight-related non-communicable diseases and maintain wholesome health. The results of this study also have implications for policy guidelines and practices on providing equitable weight management services to community members who are from ethnically diverse backgrounds.

## Conclusions

The results of this study indicate that structural and person-level factors serve as both facilitators and barriers against weight management among minoritised communities in Medway, England. On the one hand, motivations for engaging in weight management practice include social support and health benefits of weight management. On the other hand, constraining factors include systemic barriers (e.g., language barriers, non-food labelling), family commitment, migration-related changes in dietary patterns and cultural/religious norms. The results of this study could inform future studies among this population to design equitable, tailored and culturally sensitive weight management efforts to support them in adopting weight-related healthy lifestyles to protect, promote and maintain health and wellness.

### Electronic supplementary material

Below is the link to the electronic supplementary material.

Supplementary Material 1

Supplementary Material 2

## Data Availability

The datasets used and/or analysed during the current study are available from the corresponding author upon reasonable request.
