# Supplementary material for: “For me, it is for longevity and making sure I am fit and around for my children”: exploring motivations and barriers for weight management among minoritised communities in Medway, England
Source: BMC Public Health. 2024 Mar 13;24:796. doi: 10.1186/s12889-024-18281-8 (PMC10938650; doi:10.1186/s12889-024-18281-8)
Supplement: Supplementary file 1 — Supplementary Material 1 [file 12889_2024_18281_MOESM1_ESM.pdf]

Appendix VII: Interview Schedule and Interview Questions

Teesside University

Teesside University is sponsoring this project for the purposes of research governance

Title: Exploring the barriers and facilitators to access and uptake of Tier 2 weight management programmes for adult black and minority ethnic communities

Student: Jennifer Teke

Supervisors; Prof. Louisa Ells, Dr Lawrence Nnyanzi, Dr Emma Giles, (Teesside University), Mr Scott Elliott (Medway Council)

INTERVIEW SCHEDULE AND INTERVIEW QUESTIONS

- Introduce who I am and why I am doing this research
- **Discuss the aims of the interview**: To get a detailed understanding of your views on the barriers and facilitators to accessing local weight management programmes, and how best I can support you to take up weight management Intervention programmes should you be concerned with your weight.

- **Discuss practicalities of the interview**: The interview will last for up to 60 minutes but will also depend on how much you want to tell me about your weight management journey. You will be asked some questions, and it is important to remember there are no right or wrong answers. It is more important that you tell me what you really think. You do not have to answer any questions that you do not want to – just let me know and I will move on to the next question.

- **Audio-recording**: As mentioned in your information sheet, the interview will be audio-recorded. You can stop the interview at any time without giving me a reason. If you decide to withdraw your interview, you have two weeks from today (date insert) to let me know by quoting your unique identification code which can be found on the top of your participant information sheet.

- **Confidentiality and anonymity**: Some of what you say will be used in a research report, but your name will never be used. Everything that you say in the interview will be treated as completely confidential.

- The only time I might have to tell anyone is if you give disclose any risk of harm to your safety or the safety of someone else

- If at any point during the interview, you feel upset or uncomfortable, let me know, and we can stop.

- If after the interview if you would like to speak to someone about the issues discussed, your weight management service provider will be happy to speak to you.

- The fact that you have turned up now or contacted me does not mean you have to take part, you can still decide not to take part without giving any reason.

- Are you ok to continue?

INTERVIEW QUESTIONS

Section 1: Understanding of Weight Management

1. Have you ever been concerned about your weight?
    a. If yes, can you talk me through this?
2. What does weight management mean to you?
3. Are you currently trying to lose weight?
    a. How long have you been trying to lose weight?
    b. What have you been doing to try and lose weight?
4. Have you tried losing weight in the past?
    a. What method did you use and why?

Section 2: Knowledge and Awareness of Local Weight Management Programme

1. Are you aware that Medway Council runs a multicomponent weight management clinic which includes diet, physical activity and behaviour change to support individuals lose weight?
    a. Yes
    b. No

If Yes, are you currently on the programme or have you used the service before?

a. Yes (Go to Section 3)
b. No (Skip section 3)

Section 3: Views on Weight Management services

1. What are your views on your overall health since being on the weight management programme?

2. What was your experience of the weight management programme?
    a. Prompt: positive experiences?
    b. Prompt: negative experiences?

3. What aspects of the weight management program have been useful to you? Why?
4. Have you been able to attend all your appointments?
5. Prompt: If not, why?
6. How have you used the advice you were given on the weight management programme?
    a. If not, why not?
    b. Any elements you have not used? Why?

Section 4: Exploring Facilitators

1. Were you aware of the weight management programme?
2. What would motivate you to sign up for the weight management programme?
3. What aspects would you like to be covered in such a programme?
4. How would you describe your current level of motivation to start a weight management programme?
5. What might impact on this motivation?
6. What benefits would you want to see if you joined a weight management programme?

Section 5: Barriers to uptake

5a: For individuals who have participated in the weight Management Programme

1. Have you experienced any problems in participating in the weight management programme?
2. Do you think there are any challenges for participating in the programme?
    a. If yes, what are they?
    b. Why do you think they are challenges?
3. What aspects of the weight management programme would or have been most difficult for you?
    a. Why do you think there are difficult?

4. Are there any issues that affect your participation in the weight management programme?
    a. Prompt: family reasons
    b. Prompt: work reasons
    c. Prompt: community reasons
    d. Other: specify

5b: For Individuals who have not participated in the weight management programme

1. Do you have any challenges taking up the weight management programme?
    a. If yes, what are they?
    b. Why do you think they are challenges?
2. Are there any issues that affect your participation in the weight management programme?
    a. Prompt: family reasons
    b. Prompt: work reasons
    c. Prompt: community reasons
    d. Other: specify

Section 6: BME Specific questions (For those that have participated in the weight management programme)

1. Do you think weight management programmes are tailored sufficiently to meet your needs? Why/why not?
2. Is there anything missing in the programme which is useful for BME groups?
3. Is there anything in the programme that is particularly useful for BME groups?

Section 7: Improving Access and Uptake

1. How do you think health care professionals can make it easier for you and others from your background (state ethnicity as appropriate) * to get into the weight management programme?
2. What would you tell your friends and families about getting into the weight management programme?
   Prompt: How would you encourage them?
   Prompt: Benefits?
3. Is there anything else you want to tell me about getting into the weight management programme?
4. How do you think we can encourage many more people take part in the weight management programme within your community?
5. How do you think we can make it easier for many more people to take part in the weight management programmes within Medway more generally?

They are all of my questions. Do you have any questions or comments for me?

Thank you for taking the time to participate in this interview today.

*For example, if the participant is from Ghana, the question will be phrased - How can health care professionals help people from Ghana engage better in weight management programmes?
